# Polarization-sensitive tunable absorber in visible and near-infrared regimes

**DOI:** 10.1038/s41598-018-30835-6

**Published:** 2018-08-17

**Authors:** Dasol Lee, Sung Yong Han, Yeonggyo Jeong, Duc Minh Nguyen, Gwanho Yoon, Jungho Mun, Jeonghoon Chae, Jae Hyuk Lee, Jong G. Ok, Gun Young Jung, Hui Joon Park, Kyunghoon Kim, Junsuk Rho

**Affiliations:** 1 Department of Mechanical Engineering, Pohang University of Science and Technology (POSTECH), Pohang, 37673 Republic of Korea; 2 Department of Energy Systems Research, Ajou University, Suwon, 16499 Republic of Korea; 3 School of Materials Science and Engineering, Gwangju Institute of Science and Technology (GIST), Gwangju, 61005 Republic of Korea; 4 Department of Chemical Engineering, Pohang University of Science and Technology (POSTECH), Pohang, 37673 Republic of Korea; 5 Department of Mechanical and Automotive Engineering, Seoul National University of Science and Technology, Seoul, 01811 Republic of Korea; 6 Department of Electrical and Computer Engineering, Ajou University, Suwon, 16499 Republic of Korea; 7 School of Mechanical Engineering, Sungkyunkwan University, Suwon, 16419 Republic of Korea; 8 Electro-Optical System Institute, Viettel Research and Development Institute - Viettel Group, Hanoi, Vietnam

## Abstract

A broadband tunable absorber is designed and fabricated. The tunable absorber is comprised of a dielectric-metal-dielectric multilayer and plasmonic grating. A large size of tunable absorber device is fabricated by nano-imprinting method. The experimental results show that over 90% absorption can be achieved within visible and near-infrared regimes. Moreover, the high absorption can be controlled by changing the polarization of incident light. This polarization-sensitive tunable absorber can have practical applications such as high-efficiency polarization detectors and transmissive polarizer.

## Introduction

Metamaterial absorbers composed of a noble metal and dielectric can be structured at subwavelength scale to have plasmon resonances from visible to near-infrared (NIR) wavelengths. Metal-based absorber designs include gratings^1–3^, nanoparticles^4,5^ and arrays of pattern^6–8^. Metamaterial-based absorbers have been demonstrated at microwave^9–11^, terahertz^12,13^ and infrared frequencies^14–16^. Many narrow- and broad-range of absorbers have been reported at various wavelength region.

Conventional metamaterial absorbers have relied on MIM resonators formed by multilayer structures with metal and dielectric including certain patterned structures^17–20^. Extensive research on metamaterial absorbers^21–25^ has been conducted on a variety of subwavelength structures by engineering structural parameters such as size, shape and array periods. Polarization sensitive optical devices present the possibility that light absorption, transmission and reflection can be actively controlled^25,26^; this trait is necessary to fundamental photonics technologies^18,27–31^. For example, designs of narrowband polarization-sensitive absorber with bottle-like and cup-like structures have been proposed and numerically demonstrated^30^, and one consisting of arrays of U-shaped resonators has been fabricated^31^. More recently, a concept of a polarization-sensitive absorber based on plasmonic gratings has been presented^32^; it can be applied as both a broadband and a narrowband absorber.

In this work, we experimentally demonstrate a polarization-sensitive tunable absorber based on plasmonic gratings that can actively control the light absorption over a broad-range of wavelength. The structure is recently proposed with a simulation demonstration, and it is the first experimental demonstration with fabrication and confirmation of this kind of tunable absorber. The device consists of a thin (8 nm) layer of chromium (Cr) stacked between two silicon dioxide (SiO_2_) layers. A large-scale gold (Au) grating with 200 nm period is patterned using nanoimprint technique. Measured absorption of transverse electric (TE) and transverse magnetic (TM) modes show distinct behavior over the wavelength range 0.5 ≤ *λ* ≤ 1.2 μm. The absorption can be actively controlled by adjusting the polarization angle, and reaches maximum when the polarization is TE mode. Experimental results agree well with simulation in both modes. The proposed absorbers are effective in managing light absorption by using polarization.

## Results

**Figure 1:**
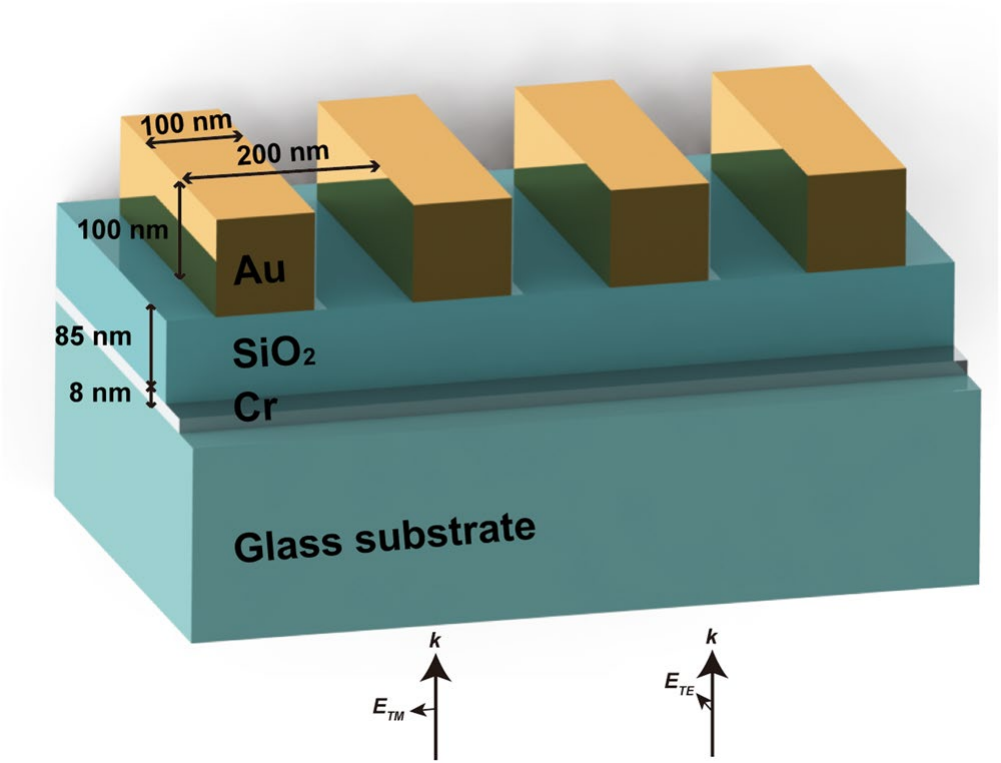
Structure of broadband tunable absorber with plasmonic grating: 8 nm Cr and 85 nm SiO_2_ layers are deposited on a glass substrate, then an Au grating with 100 nm width and 200 nm period is patterned on the SiO_2_ layer. The incident light is shone on the bottom of the structure. The absorption can be actively controlled by adjusting the polarization of incidence light.

The broadband tunable absorber with plasmonic gratings based on the multilayer structure of dielectrics and metal is described in Fig. 1. Our design for broadband tunable absorber consists of a thin Cr layer between two SiO_2_ layers and Au grating with period of 200 nm and 100 nm thickness. In the conventional structure of absorber, the top layer (Au grating) will be a reflector layer such as Au layer instead of grating. This top layer has a role to reflect the light back and makes the Cr-SiO_2_-Au structure become a resonator to absorb energy at resonant wavelength as calculated in reference^18^. For further understanding of the tunable absorber device, the impedance transform method^33^ was applied to the metal and dielectric multilayer system. The device can be considered as multilayers of metal and dielectric with layer thickness of *d*_*m*_ and *d*_*d*_. The impedance of the n^th^ layer (*Z*_n_) can be expressed as

1 $${Z}_{n}=\frac{1}{\cos \,\theta }\sqrt{\frac{{\mu }_{n}}{{\varepsilon }_{n}}}$$

where *μ*_*n*_ is the permeability of the layer, *ε*_*n*_ is its permittivity, and *θ* is the angle of incidence. *Z*(*n*) can be defined as wave impedance of an interface between the *n*^*th*^ and (*n* + 1)^*th*^ layers. Then boundary condition between each layer gives a recursion formula of the wave impedance.

2 $$Z(n)={Z}_{n}\frac{Z(n-1)-i{Z}_{n}\,\tan \,{\phi }_{n}}{{Z}_{n}-iZ(n-1)\,\tan \,{\phi }_{n}}$$

where *φ*_*n*_ = *k*_*Z*_*d* (*d* is the thickness of the layer) is the phase gain of the *n*^*th*^ layer. The reflection coefficient of the multilayer can be easily obtained from eq. (2) as

3 $$r=\frac{{Z}_{0}-{Z}_{N}}{{Z}_{0}+{Z}_{N}}$$

where *Z*_0_ and *Z*_*N*_ are characteristic impedance of the input and the output medium, respectively. When the characteristic impedance of the input and the output medium is matched (*Z*_0_ = *Z*_*N*_), the reflection becomes zero at the air/dielectric layer and transmission at Au layer is also zero. Consequently, the absorption can be calculated using reflection coefficient as *A* = 1 − *T* − *R* (*R* is reflection and *T* is transmission). We assume that real part of the Au top layer can be negligible and the Cr layer is much thinner than the optical wavelength. Periodic nature of the eq. (2) leads to multiple absorption bands as the function of *k*_*Z*_ and *d*_*d*_ when the Cr is fixed to 8 nm. The calculated absorption band shows high absorption in broad range of wavelength^18^ because the permittivity of Cr^18^ satisfies the matching impedance condition in visible to near-infrared regime.

**Figure 2:**
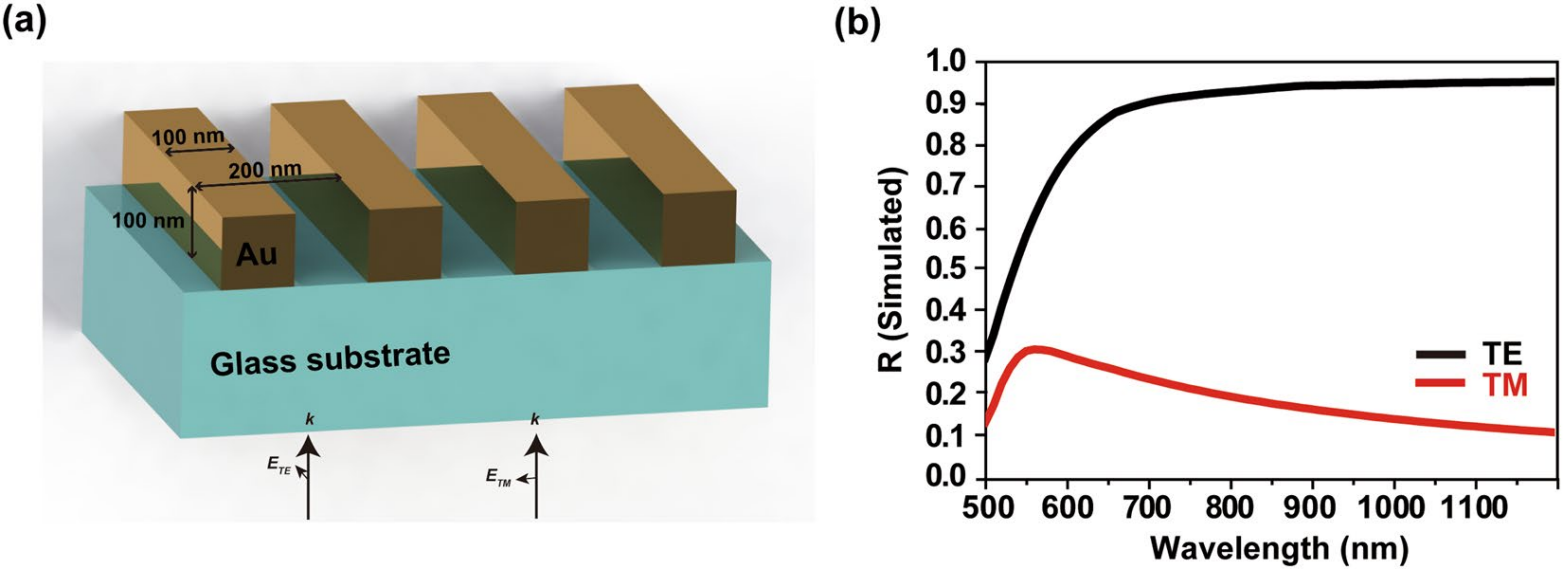
(**a**) Au grating on glass substrate. (**b**) Reflectance for TE and TM mode light under normal incidence.

In our polarization-sensitive absorber design, we added a plasmonic grating structure (Fig. 2(a)) with subwavelength scale to the last layer to break polarization degeneracy. The simulation used the Rigorous Coupled Wave Analysis (RCWA) method, which can be applied to metallic or dielectric grating structures. The TE mode and TM mode cases were simulated to predict how polarization affected absorption by the device. Due to the rapid field variation in a grating structure shorter than the wavelength of incidence light, high harmonic order is necessary to get a convergence in calculation. Two hundred spatial harmonics were considered to achieve convergence in our metal grating, and convergence in RCWA simulation was achieved. When the top layer is Au grating, this layer reflects only TE mode and transmits the TM mode for broad range of wavelength from visible to near-infrared as shown in Fig. 2(b). At TE mode, the Au grating layer will reflect all light back to the SiO_2_-Cr-SiO_2_ layers, meaning zero transmission (*T* = 0) through the top Au grating layer and form a resonant structure. According to the formula *A* = 1 − *T* − *R*, the proposed structure is then expected to act as an absorber at TE mode, inversely has low-absorption at TM mode due to a high transmission through the grating layer at this mode. Consequently, different absorption bands can be achieved by using different polarization states of incident light.

**Figure 3:**
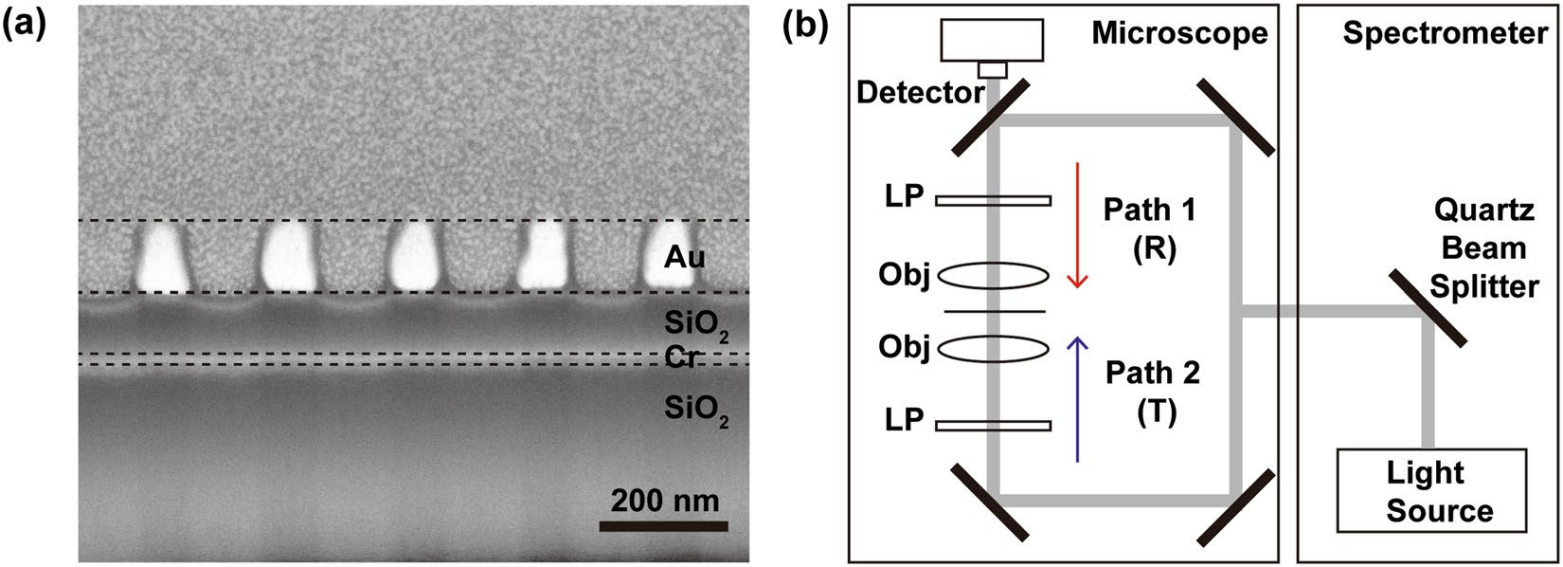
(**a**) Cross-sectional SEM image of fabricated tunable absorber. On the glass substrate, thin Cr layer and SiO_2_ were deposited using electron beam evaporation, then an Au grating with 200 nm period was patterned on the SiO_2_ layer by nanoimprint lithography. To get a clear SEM image, platinum (Pt) is deposited on the Au grating and cut using dual-beam focused ion beam milling system (Helios Nanolab G3 CX, FEI). (**b**) Measurement setup for broadband tunable absorber. A microscope connected to the spectrometer is used to capture transmittance *T* and reflectance *R* spectra. The polarization of incidence light is controlled using a linear polarizer.

The tunable absorber is fabricated and cross-sectional SEM image is shown in Fig. 3(a). An Au grating with 200 nm period, 100 nm thickness in centimeter scale was patterned on the SiO_2_-Cr-SiO_2_ layer. The fabricated tunable absorber device was measured using a microscope connected to an FT-IR spectrometer (Vertex 70 and Hyperion 2000, Bruker) including a silicon detector and a quartz beam splitter as shown in Fig. 3(b). A white light tungsten lamp was used and a linear polarizer was put into the illumination light path to input 0° and 90° polarizations. Transmitted and reflected light were collected with appropriate incidence light direction and polarization. Reflectance measurement was calibrated using a broadband mirror with an average reflectance of 99% in the visible to near infrared (Vis-NIR) range. The measured transmittance T and reflectance R were used to calculate absorption *A* = 1 − *T* − *R*.

**Figure 4:**
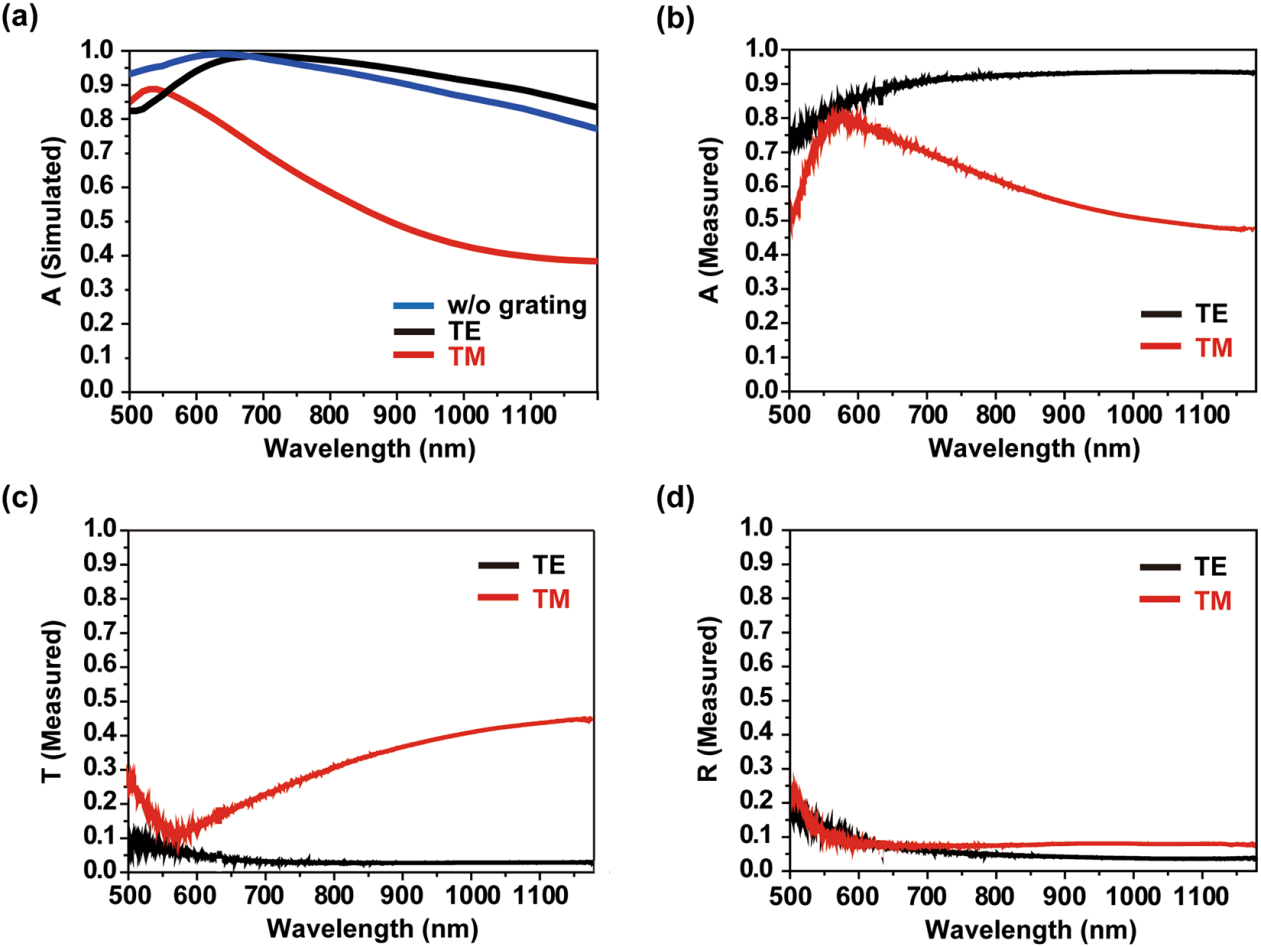
(**a**) Calculated absorption spectra of the tunable absorber. Absorption spectra in TE mode, TM mode and the case without grating (200 nm Au layer) is expressed as black line, red line and blue line, respectively. (**b**) Measured absorption spectrum from broadband tunable absorber. The absorption shows different properties with different polarization angle. Simulated and measured results show quite similar tendency and have broadband high absorption in the broad range of wavelength. (**c**) Measured transmittance spectrum and (**d**) measured reflectance spectrum with different polarizations.

A polarization-sensitive tunable absorber was achieved by combining an impedance-matched multilayer and a grating. First, numerical investigation of the broadband tunable absorber was conducted for comparison with experiment. The permittivities of Au^34^ and Cr^35^ are used for numerical RCWA simulation. In simulations, the TE waves exhibited high absorption over 0.5 ≤ *λ* ≤ 1.2 μm, whereas absorption of the TM mode diminished rapidly and was <40% in the near-infrared range as shown in Fig. 4(a). The measured spectra in Fig. 4(b) were similar to the simulation results; absorption was >90% in TE mode in visible and near infrared wavelength, but decreased rapidly in TM mode as wavelength increased. The underlying mechanism of the polarization dependent absorption is the polarization-sensitive reflection from the grating. In our design and fabrication, Au grating structure has long length in centimeter scale that is enough to avoid a localized plasmon mode in TE mode. The well-made long scale Au grating acts as a reflector layer only in TE mode and high absorption is achieved at TE mode. The measured transmittance spectrum shows that only the TM mode can pass through the grating structure, and that TE mode light reflects back to the multilayer as shown in Fig. 4(c). In contrast, because of the impedance matching, reflection is negligible in both TE and TM modes as shown in Fig. 4(d). Even though the measured absorption is above 90% for a broad range of wavelength over 0.5 ≤ *λ* ≤ 1.2 μm, there can be an experimental errors which make the absorption lower; (i) fabrication of grating, (ii) thickness of dielectric and Cr layer after e-beam evaporation having possibility of experimental errors and (iii) number of grating limited in scale which should be infinite in theory. Thus, precise fabrication and measurement is required for absorber having high absorption.

**Figure 5:**
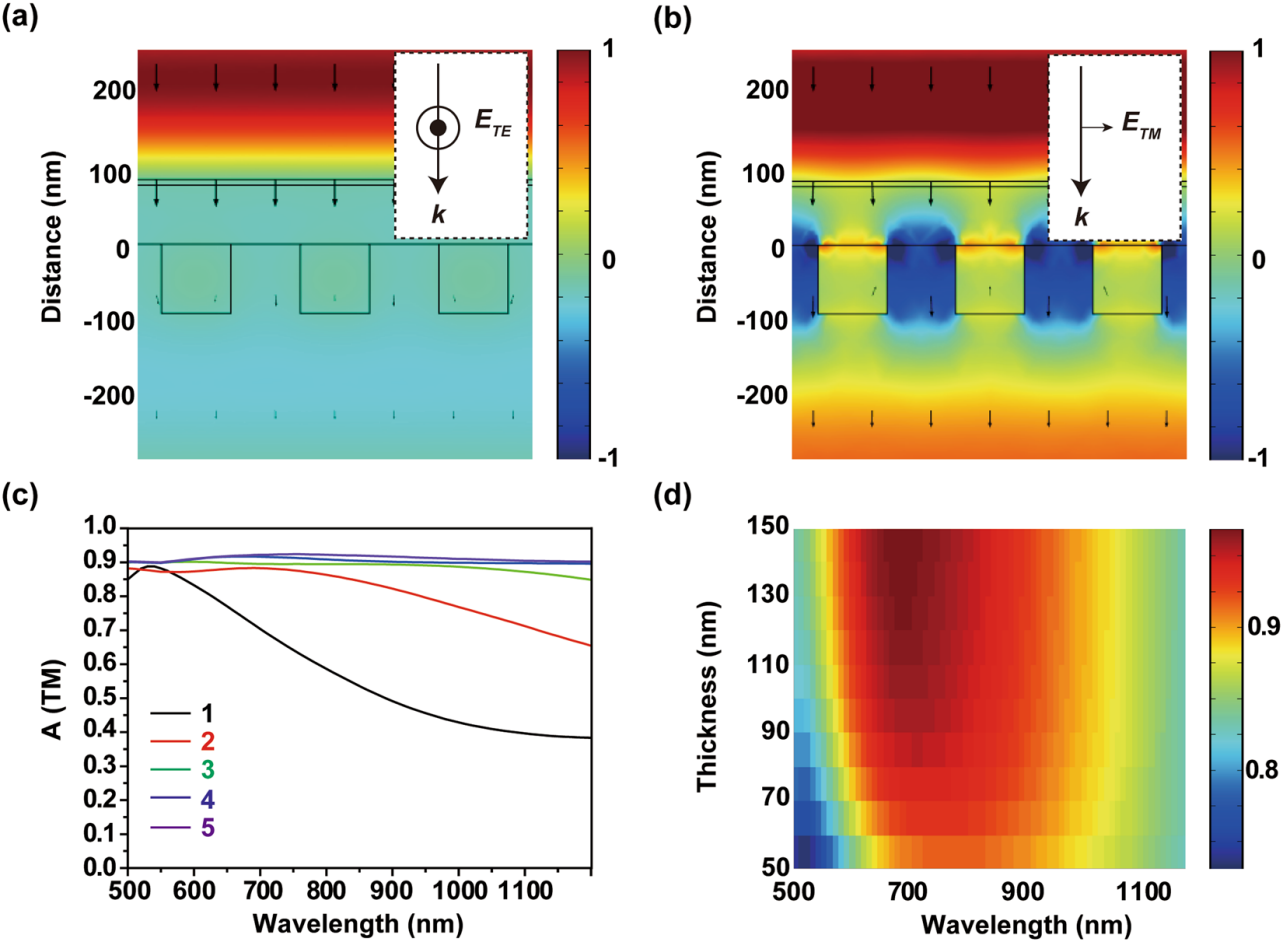
(**a**,**b**) Electric field distribution on the x-z plane at *λ* = 800 nm and the time-averaged power flow $\overrightarrow{P}$ (arrows) for (**a**) TE mode and (**b**) TM mode. (**c**) Absorption spectra of Cr-SiO_2_ absorbers with different numbers of layers. (**d**) Absorption sensitivity as a function of a grating thickness under illumination by light in TE mode.

Electric field distributions were obtained at *λ* = 800 nm under normal incidence illumination in Fig. 5(a,b). In TE mode, the impedance matching by the absorber can be observed using the electric field distribution. In the electric field distribution, the wavefront of the incident light does not show any distortion as it propagates toward the dielectric layer; this result means that the absorber reflected no light. The Cr layer induces the absorption of the incident light as shown in Fig. 5(a). The time-averaged power flow shows constant intensity in the substrate but decays rapidly below Cr layer. However, in TM mode, the light can penetrate the absorber structure due to the polarization-selectivity of the grating. In TM mode, light penetrates the grating structure and absorption is low as shown in Fig. 5(b). These results show that highly sensitive absorption control can be achieved by controlling the polarization of the incident light.

The purpose of this work is to demonstrate a controllable broadband tunable absorber, and significant parameters to control the absorption regime are considered. Calculated absorption spectra at 0.5 ≤ *λ* ≤ 1.2 μm changed when additional Cr-SiO_2_ layers were added below the grating structure as shown in Fig. 5(c). The absorption became polarization insensitive when pairs of Cr-SiO_2_ layers were added. Due to the improvement of absorption which can be reached up to the longer wavelength, the absorption looks insensitive to polarization in the target Vis-IR region. Thus, one pair of Cr-SiO_2_ layer between the substrate and grating structure is the optimal design for a polarization-sensitive tunable absorber within this wavelength region. Grating thickness was also considered under TE mode to investigate the tolerance influenced by error in fabrication thickness. Calculations suggest that the absorption sensitivity is nearly unaffected by variation in grating thickness 100 nm to 150 nm as shown in Fig. 5(d).

In summary, we have first demonstrated a polarization-sensitive tunable absorber based on a plasmonic grating structure as the reflector layer in broadband wavelength range 0.5 ≤ *λ* ≤ 1.2 μm. Light absorption by this absorber can be adjusted by changing the polarization of incident light. Light absorption was >90% in a broad wavelength band in TE mode, but diminished when the polarization was changed to TM mode. This phenomenon can be exploited to realize a broadband optical switching system controlled by polarization. The broadband absorber has good absorption with optimized number of layers, and a wide range of grating thickness. Many broadband absorber have shown useful applications in thermo-photovoltaics^36–38^. Due to the polarization sensitivity of our device, we expect that real applications of this absorber with plasmonic grating can inspire a new concept of control of light-absorbing devices with applications in polarization detectors^30,31^ and transmissive polarizer^39,40^ at visible and near-infrared regime.

## Methods

### Sample Fabrication

SiO_2_ glass substrate was first covered with a Cr layer (8 nm) and a SiO_2_ dielectric layer (85 nm) by using electron beam evaporation. The deposition rates were 0.1 nm/s in both cases. After deposition of these layers, a centimeter scale of Au grating pattern with 200 nm period was fabricated using nanoimprint lithography using a SiO_2_ mold on poly (methyl methacrylate) (PMMA) resist at a pressure of 50 bar, and temperature of 170 °C for 7 min. The imprinted grating structures were cooled and demolded, then Cr was selectively deposited on each sidewall by angled deposition to induce the undercut structures during subsequent O_2_ reactive ion etching (RIE); this process facilitated the lift-off process and controlled the line-width of the resultant metal grating. O_2_ RIE was performed using 10 sccm of O_2_ at chamber pressure of 40 mTorr and bias power of 40 W. Then 100 nm thickness of Au was deposited using an electron-beam evaporator, and the mold was lifted off.

## Data Availability

All data generated or analyzed during this study are included in this published article.
